# Biological Properties, Mineral Composition, and Health-Promoting Potential of Tiger Nut Tubers (*Cyperus esculentus* L.) as a Novel and Underutilized Food Source

**DOI:** 10.3390/foods15020191

**Published:** 2026-01-06

**Authors:** Zuzana Knazicka, Tunde Jurikova, Eva Kovacikova, Katarina Fatrcova-Sramkova, Vladimira Bella, Branislav Galik, Klaudia Tomasova, Liliana Hnatova, Ivona Janco, Dominika Lenicka, Martyna Błaszczyk-Altman, Eva Ivanisova, Sona Skrovankova, Martin Prcik, Jiri Mlcek

**Affiliations:** 1 Institute of Nutrition and Genomics, Faculty of Agrobiology and Food Resources, Slovak University of Agriculture in Nitra, Tr. Andreja Hlinku 2, 949 76 Nitra, Slovakia; zuzana.knazicka@uniag.sk (Z.K.); eva.kovacikova@uniag.sk (E.K.); branislav.galik@uniag.sk (B.G.); xtomasovak@uniag.sk (K.T.); xhnatoval@uniag.sk (L.H.); 2 Institute for Teacher Training, Faculty of Central European Studies, Constantine the Philosopher University in Nitra, Drazovska 4, 949 74 Nitra, Slovakia; tjurikova@ukf.sk; 3 AgroBioTech Research Centre, Slovak University of Agriculture in Nitra, Tr. Andreja Hlinku 2, 949 76 Nitra, Slovakia; vladimira.bella@uniag.sk (V.B.); ivona.janco@uniag.sk (I.J.); eva.ivanisova@uniag.sk (E.I.); 4 Institute of Plant Production, Faculty of Agrobiology and Food Resources, Slovak University of Agriculture in Nitra, Tr. Andreja Hlinku 2, 949 76 Nitra, Slovakia; dominika.lenicka@uniag.sk; 5 Institute of Biology and Earth Sciences, Faculty of Exact and Natural Sciences, University of the National Education Commission, ul. Podchorążych 2, 30-084 Krakow, Poland; martyna.blaszczyk-altman@uken.krakow.pl; 6 Institute of Food Sciences, Faculty of Biotechnology and Food Sciences, Slovak University of Agriculture in Nitra, Tr. Andreja Hlinku 2, 949 76 Nitra, Slovakia; 7 Department of Food Analysis and Chemistry, Faculty of Technology, Tomas Bata University in Zlin, Vavreckova 5669, 760 01 Zlin, Czech Republic; skrovankova@utb.cz; 8 Institute of Sustainable Regional and Local Development, Faculty of European Studies and Regional Development, Slovak University of Agriculture in Nitra, Tr. Andreja Hlinku 2, 949 76 Nitra, Slovakia; martin.prcik@uniag.sk

**Keywords:** *Cyperus esculentus* L., biological properties, fatty acid composition, mineral profile, health risk assessment

## Abstract

Tiger nut (*Cyperus esculentus* L.) is a relatively neglected tuber crop with notable nutritional, functional, and ecological value. The primary objective of this study was to evaluate the biological properties and selected nutritional parameters of tiger nut tubers and oil, including antioxidant activity, total phenolic content (TPC), fatty acid (FA) profile, health-related lipid indices, and mineral composition. Methods: Natural and peeled tiger nut tubers, as well as commercially available tiger nut oil (yellow variety, Valencia, Spain), were analyzed. Antioxidant activity was measured spectrophotometrically using the DPPH method. The content of TPC was determined using the Folin–Ciocalteu assay. Fatty acid composition was analyzed by gas chromatography coupled with flame ionization detection, and these data were used to calculate the PUFA/SFA (P/S) ratio, atherogenicity (AI), thrombogenicity (TI) index, and hypocholesterolemic/hypercholesterolemic (h/H) ratio. Macro- and microelement contents were quantified using inductively coupled plasma optical emission spectrometry. Estimated daily intake (EDI), target hazard quotient (THQ), and total THQ (TTHQ) were calculated to assess potential health risks. Results: Natural tiger nut tubers exhibited substantially higher antioxidant activity and TPC compared to peeled tubers, suggesting that the peel is the primary reservoir of phenolic compounds. Strong antioxidant activity was observed in tiger nut oil (64.82 ± 2.59 mg TEAC/L). Oleic acid (C18:1cis *n*-9) was identified as the predominant FA across all samples, thus contributing positively to favorable health lipid indices (P/S > 0.50, low AI and TI, high h/H ratio). Potassium was the most abundant macroelement in natural and peeled tiger nut tubers. The overall trend of microelement levels in these samples was as follows: Al > Fe > Zn > Cu > Sr > Mn > Li > Ba > Se > As > Cr. All THQ and TTHQ values were below 1, indicating no appreciable health risk associated with consumption. Conclusions: These findings support the use of tiger nuts as a functionally valuable ingredient in health-oriented food products.

## 1. Introduction

Human nutrition and food systems are at the center of today’s most pressing global challenges, including the rising incidence of diet-related diseases, climate change, biodiversity loss, and the depletion of natural resources. Against this background, it is crucial to utilize alternative plant-based food sources with high nutritional and ecological value and minimal health risk is crucial. Moreover, tuber plants are the second most important source of foods rich in natural antioxidants [1]. Tiger nut tubers (*Cyperus esculentus* L.) have been cultivated for centuries as a rich source of bioactive compounds and have the potential to play a significant role in addressing current challenges related to food scarcity.

*Cyperus esculentus* L. is a monocotyledonous, edible perennial plant belonging to the grass family *Cyperaceae* [2,3,4]. It has been widely cultivated as a cosmopolitan plant, particularly in Asia (India, China), West African countries (Egypt, northern Nigeria, Niger, Mali, Senegal, Ivory Coast, Ghana, and Togo), and several European and adjacent countries (Greece, France, Italy, Portugal, Turkey, Israel, the Netherlands, and the United Kingdom) [5,6,7,8,9]. It is especially prominent in Valencia (Spain), where it has been cultivated using organic farming practices [10].

Globally, *Cyperus esculentus* L. is known by various names, including chufa, subterranean walnut, earth almond, rush nut, edible galingale, yellow nutsedge, yellow nutgrass, imunu, Zulu nuts, ayaya, edible rush, ofi, and akiausa [11,12,13]. Three main varieties (black, red, and brown/yellow) are recognized. Among these, the yellow variety is the most favored due to its fleshier texture, appealing color, and larger size [4,8,14,15]. The variation in color among the different varieties is attributed to enzymatic browning and multiple chemical changes that occur during the dehydration process. For this reason, some authors suggest that the yellow and brown varieties of tiger nuts could potentially represent the same type [16,17]. According to Emurotu [18], the yellow and brown varieties can be used interchangeably. However, there are significant differences in their mineral composition, with the yellow variety exhibiting elevated concentrations of mineral constituents.

*Cyperus esculentus* L. can be characterized as a high-quality oilseed crop that has attracted considerable interest in the food and nutraceutical industries [13,19]. Tiger nut tubers are processed into various products, including horchata, snacks, beverages, and gluten-free bread. Although they have a rich history of being used as food and feed sources tiger nuts and their constituents still require further investigation, particularly regarding their functional properties, potential modifications, and advanced processing techniques. The nutritional composition of tiger nuts depends on several factors, including variety, soil and environmental conditions, cultivation methods, and notably, storage practices [20].

In terms of energy, tiger nut tubers provide 400–414 kcal/100 g [21]. They contain high amounts of carbohydrates (≈47%) [14,17], especially starch (14–37%) [22,23], fats (20–38%), and a low protein content (6.10–9.70%) [21,24,25,26], as well as dietary fiber (8–9%) [27]. *Cyperus esculentus* L. is a rich source of both saturated and unsaturated fatty acids (FAs), containing high levels of oleic acid (56–85%), along with smaller amounts of palmitic (10–20%), linoleic (8–12%), and stearic acid (0.30–5.30%) [17]. The presence of dietary fibers plays a significant role in alleviating digestive problems and obesity [28]. Although tuberous vegetables are not typically acknowledged for their protein levels, four protein fractions have been identified, with glutelin being the most abundant (47.50%), followed by albumin (31.80%), globulin (4.70%), and prolamine (3.80%) [29]. Tiger nut tubers possess a substantial concentration of polyphenolic compounds, alkaloids, steroids, terpenoids, flavones, saponins, tannins, and other phytoprotective compounds that provide a broad spectrum of health advantages [15,21,30,31]. Furthermore, their significant antioxidant potential, related to their flavonoid composition, may provide natural protection against free radicals [32]. Numerous studies indicate that tiger nut tubers are rich in mineral elements such as potassium (K), calcium (Ca), magnesium (Mg), iron (Fe), copper (Cu), zinc (Zn), and manganese (Mn) [14,33,34,35].

Vegetable oils are a crucial component of the human diet. The distinctive flavor of tiger nut oil makes it an appealing culinary ingredient worthy of further attention. A limited number of investigations have examined its potential as an edible oil [13]. Tiger nut oil has a distinctive sweet, nutty aroma and a golden color, characteristics attributed to its high content of pigments and phenolic compounds [36]. Its FA composition closely resembles that of olive oil, with both oils being rich in monounsaturated fatty acids (MUFAs) and containing only small amounts of polyunsaturated fatty acids (PUFAs) [13]. The high oleic acid content in tiger nut oil contributes to its oxidative stability, as confirmed by Ezeh et al. [37] and Sobhani et al. [38], supporting its potential as an alternative or supplementary source of high-quality cooking oil. Oils rich in oleic acid should be consumed as part of the daily fat intake, alongside a sufficient intake of PUFA, as the consumption of oleic acid has been correlated with a reduced risk of cardiovascular diseases [39]. Most tiger nut research papers deal with the extraction or application of tiger nut oil [40,41,42]. Tiger nut oil consists of 64–73.30% oleic acid and 11–15.50% linoleic acid, which is comparable to olive oil (55–83% oleic acid and 3.50–21% linoleic acid) and significantly exceeds the oil content in soybean, peanut, and rapeseed oils [43].

The underground tubers of *Cyperus esculentus* L. play a significant role in health protection, exhibiting preventive and therapeutic effects, and offering potential for supportive therapies in metabolic and lifestyle-related diseases [33,44]. They may help reduce the risk of hypercholesterolemia and type 2 *diabetes mellitus* [45]. Tiger nut consumption is believed to improve blood circulation and may help prevent cardiovascular and gastrointestinal diseases, reduce hypertension, and lower the risk of colorectal cancer [46]. They are also suitable for gluten-free flour blends, making them beneficial for individuals with celiac disease. Several studies have confirmed the antibacterial activity of these tubers [47,48].

The primary aim of the current research was to examine antioxidant activity, total phenolic content (TPC), FA composition, and health-related lipid indices, as well as mineral composition in natural and peeled tiger nut tubers and oil. In addition, the study also explored the potential application of these properties for disease prevention. Based on the measured concentrations of elements, the estimated daily intake (EDI), target hazard quotient (THQ), and total target hazard quotient (TTHQ) were calculated to assess potential health risks associated with tiger nut consumption. Furthermore, this research highlights the underutilized status of tiger nut tubers, emphasizing their potential to diversify food sources and contribute to global food security. Recognized as a fundamental component of a diet that enhances health and sustains balance; the study underscores the need for greater scientific attention, increased consumer awareness, and stronger engagement from the food industry.

## 2. Materials and Methods

### 2.1. Experimental Material

The samples were acquired from a specialized company that imports tubers of *Cyperus esculentus* L. from Valencia (Spain) and focuses on products made from tiger nuts (yellow varieties). Peeled tiger nuts were obtained by mechanically scraping the outer surface of the tubers, thereby removing part of their skin. Oil samples were packaged in amber bottles with cardboard covers to prevent exposure to light and temperature changes. They reflect the actual state of bulk oils in commercial trade, providing an accurate basis for evaluation. The oils were kept in a low-temperature environment (10 °C) and protected from light exposure.

### 2.2. Extract for Analysis of Antioxidant Activity and Total Phenolic Content

Extracts of natural and peeled tiger nut tubers were prepared by adding 1.0 mL of extraction agent (methanol:H_2_O:acetic acid in a ratio of 80:18:2) to 20 mg of the sample. The specimens underwent homogenization and sonication (5 min, 50 °C). Subsequent to centrifugation (15,616× *g* for 10 min, 20 °C) (Hettich Rotina 380, Tuttlingen, Germany), the supernatant was carefully transferred into clean microtubes. An additional 1.0 mL of extraction solvent was added to the pellet, and the entire procedure was repeated [49]. Each sample was prepared in two technical replicates.

### 2.3. Determination of Antioxidant Activity

The antioxidant activity was determined spectrophotometrically according to the method of Brand-Williams et al. [50] adapted for use with 96-well microplates [49]. Briefly, to prepare the 2,2-diphenyl-l-picrylhydrazyl (DPPH•) reagent (Merck KGaA, Darmstadt, Germany), 25 mg was dissolved in 100 mL of 96% ethanol (Centralchem Ltd., Bratislava, Slovakia) and adjusted to an absorbance of 0.7. In a 96 well-microplate, 25 μL of the sample, blank, or standard (in triplicate) was added, followed by 180 μL of DPPH• solution. After resting for 10 min in the dark at room temperature (23 °C) with simultaneous shaking at 500 rpm, the absorbance of the samples was measured using a DYNARead microplate photometer (Dynex Technologies s.r.o., Praque, Czech Republic) at a wavelength of 515 nm. The DPPH radical scavenging activity was expressed as Trolox equivalent antioxidant capacity (TEAC) in mg per g of dry weight (DW) or mg per L, respectively, calculated using a Trolox standard curve ((±)-6-hydroxy-2,5,7,8-tetramethylchroman-2-carboxylic acid; Sigma-Aldrich, Schnelldorf, Germany; concentration range 0–100 mg/L; R^2^ = 0.9969).

### 2.4. Determination of Total Phenolic Content

To evaluate the TPC, the Folin–Ciocalteu method described by Xiong et al. [51] was used, with slight modifications for use with 96-well microplates [49]. Briefly, in a 96-well microplate, 50 μL of distilled water was added, followed by 25 μL of the sample or standard (in triplicate) and 25 μL of Folin–Ciocalteu reagent (Merck KGaA, Darmstadt, Germany). After 6 min, 100 μL of 7.5% (*w*/*v*) sodium carbonate solution (Centralchem Ltd., Bratislava, Slovakia) was added to all wells. The plate was then left in the dark for 90 min at room temperature (23 °C). Absorbance was measured using a UV-Vis plate reader (Glomax Multi+, Promega Corp., Madison, WI, USA) at 765 nm. The TPC was estimated using a gallic acid calibration curve (0–500 mg/L; R^2^ = 0.9749) and expressed as gallic acid equivalent (GAE) in mg per g of DW or mg per L, respectively.

### 2.5. Determination of Fatty Acid Composition

The procedure for the determination of FA composition was described by Knazicka et al. [52]. The fatty acid methyl esters (FAMEs) were extracted with petroleum ether and analyzed using gas chromatography (GC) employing a flame ionization detector (FID) on an Agilent 6890A (Agilent Technologies, Santa Clara, CA, USA) configured with a multi-mode injector. An analytical DB-23 column (Agilent Technologies 122-2361; dimensions of 60 m × 250 μm × 0.15 μm) was utilized for the chromatographic separation. 37-component standard mixture (Supelco 47885-U; Sigma-Aldrich, Laramie, WY, USA) was applied for column calibration. Helium was used as the carrier gas at a head pressure of 2.225 mL/min at a constant flow. For each analysis, 1.0 μL of the sample was injected. The inlet and detector temperatures were set at 280 °C. Peaks were identified by comparing retention times with those of a mixture of external standard methyl esters. The FAs were calculated as the percentage of the sum of FAs using Agilent OpenLab ChemStation software (OpenLab CDS ChemStation Edition B.04.01). Each sample was analyzed in duplicate.

### 2.6. Calculation of Health Lipid Indices

Comprehensive analyses of SFA, MUFA, and PUFA contents, alongside the PUFA/SFA (P/S) ratio, as well as the atherogenic index (AI), thrombogenic index (TI) [53], and the hypocholesterolemic/hypercholesterolemic ratio (h/H) [54,55], were conducted. The health indices AI, TI, and h/H were employed to assess the nutritional quality of the FA composition present in natural and peeled tiger nut tubers, as well as the extracted oil. The AI and TI indices quantitatively represent the impact of specific FAs on cardiovascular risk factors, while the h/H ratio specifies the functional consequences of FAs on cholesterol metabolism [53]. They were calculated according to the following equations:

(1) Index of atherogenicity according to Ulbricht and Southgate [53]:

$$\mathrm{AI}\;=\frac{C12:0+(4\;\times \;C14:0)+C16:0}{\Sigma \mathrm{UFA}}$$

(2) Index of thrombogenicity according to Ulbricht and Southgate [53]:

$$\mathrm{TI}=\frac{\left(C14:0+C16:0+C18:0\right)}{\left[(0.5\times \Sigma MUFA)+(0.5\times \Sigma n-6\;PUFA)+(3\times \Sigma n-3\;PUFA)+(n-3/n-6)\right]}$$

(3) hypocholesterolemic/hypercholesterolemic ratio according to Fernández et al. [55]:

$$h/H=\frac{\left(C18:1cisn-9+C18:2cisn-6+C18:3n-3+C20:1n-9\right)}{\left(C14:0+C16:0\right)}$$

### 2.7. Determination of Mineral Composition

Macro- and microelement contents in the samples were subjected to analysis as previously described by Árvay et al. [56]. Each sample was analyzed in triplicate using inductively coupled plasma optical emission spectrometry (ICP-OES; Agilent ICP-OES 720, Agilent Technologies Inc., Santa Clara, CA, USA) with an axial plasma configuration, and an SPS-3 autosampler (Agilent Technologies GmbH, Basel, Switzerland). Suitable standards for every mineral element were prepared within the concentration range of elements contained in the sample. The selected elements were determined under standard conditions: power (0.90 kW), replicate read time (3 s), instrument stabilization (20 s), sample uptake delay (25 s), pump rate (15.0 rpm), rinse time (20 s), and CCD detector temperature (−35 °C). Gas flow parameters of the ICP-OES included plasma of 15 L/min, auxiliary of 1.50 L/min, and nebulizer of 1.0 L/min. The following elements were determined in the prepared solutions: Ag (328.068 nm), Al (167.019 nm), As (188.980 nm), Ba (455.403 nm), Ca (315.887 nm), Cd (226.502 nm), Co (228.615 nm), Cr (267.716 nm), Cu (324.754 nm), Fe (234.350 nm), K (766.491 nm), Li (670.783 nm), Mg (383.829 nm), Mn (257.610 nm), Na (589.592 nm), Ni (231.604 nm), Pb (220.353 nm), Sb (206.834 nm), Se (196.026 nm), Sr (407.771 nm), and Zn (206.200 nm). In the study, Multielement Standard Solution V for ICP (Sigma-Aldrich Production, GmbH, Buchs, Switzerland) was used. Argon and carbon were employed as internal standard elements. The integrity of the comprehensive methodology was substantiated through the utilization of certified reference material (CRM) ERMR-CE278k (muscle tissue; IRMM, Geel, Belgium).

### 2.8. Health Risk Assessment

A thorough health risk assessment was performed to explore the possible adverse effects associated with the consumption of tiger nut tubers. The evaluation was based on risk indicators, including the estimated daily intake (EDI), which represents the quantity of a specific substance to which an individual may be exposed daily through diverse sources (e.g., nuts). Subsequently, utilizing the obtained EDI values, the target hazard quotient (THQ) was determined. The EDI was derived using the following equation:

$$\mathrm{EDI}\;=\frac{C\times IR}{BW}$$

where EDI represents the calculated estimated daily intake (mg/kg/body weight/day), C reflects the average concentration of the element in tiger nuts—including both natural and peeled tubers (mg/g DW), IR indicates the daily ingestion rate (g/person/day), and BW is the average body weight (kg) [57]. According to a study by Jenab et al. [58], conducted as part of the European Prospective Investigation into Cancer and Nutrition (EPIC), the estimated average intake of all tree nuts in the Spanish population was 2.99 g/day. For Slovakia, specific data on the daily ingestion rate are currently not available; however, only food-based dietary guidelines recommendations for nuts and seeds are provided. A “handful per day” is commonly used as a practical guide for consumers [59]. It is widely proposed that this corresponds to a recommended portion of 30 g [60]. The average adult body weight in Europe was set at 70 kg [61].

The target hazard quotient (THQ) serves as a metric for evaluating the potential health risk to consumers due to metal-polluted tiger nut tuber consumption, using the oral reference dose (RfD). A THQ < 1 suggests that the ingestion of the specified tiger nut tubers provides a health advantage, thus demonstrating a certain degree of safety for consumers; conversely, a THQ > 1 indicates a potential for adverse health risks. The THQ was calculated using the following equation:

$$\mathrm{THQ}\;=\;\frac{EDI}{RfD}$$

where EDI is the estimated daily intake (mg/kg body weight/day) and RfD is the oral reference dose (mg/kg/day) [57]. According to the United States Environmental Protection Agency (US EPA) [62], the RfDs are as follows (mg/kg/day): Al = 1.0, Ba = 0.20, Cu = 0.04, Fe = 0.70, Li = 0.002, Mn = 0.14, Se = 0.005, Sr = 0.60, Zn = 0.30. RfDs for macroelements such as Ca, Mg, Na, and K have not been established by the US EPA, as these elements are required for normal physiological function.

The total target hazard quotient (TTHQ) was calculated as the sum of the individual THQs for all analyzed elements.

### 2.9. Statistical Analysis

All obtained data were statistically evaluated using GraphPad Prism 8.0.1 (GraphPad Software Incorporated, San Diego, CA, USA). One-way analysis of variance (ANOVA), followed by Tukey’s multiple comparison test, or the Kruskal–Wallis test, followed by Dunn’s multiple comparison test, were applied to determine significant differences, with significance thresholds set at *p* < 0.001, *p* < 0.01, and *p* < 0.05.

## 3. Results and Discussion

### 3.1. Evaluation of Antioxidant Activity and Total Phenolic Contents

Tiger nut tubers possess significant antioxidant activity, predominantly attributed to their elevated concentration of phenolic compounds such as quercetin, myricetin, and other bioactive constituents, along with vitamins C and E [63,64]. Previous studies have consistently demonstrated the antioxidant activity of tiger nut tubers and by-products using a range of in vitro assays, including DPPH, ABTS (radical cation decolorization assay) and FRAP (ferric reducing antioxidant power), in order to capture different radical-scavenging mechanisms [65,66,67]. Our results indicate that antioxidant activity is strongly dependent on both the matrix and the degree of processing. Significant differences were observed among natural and peeled tubers, as well as tiger nut oil (Table 1). Natural Spanish tiger nut tubers exhibited substantially higher antioxidant activity (2.77 ± 0.201 mg TEAC/g DW) compared to peeled tubers (1.49 ± 0.270 mg TEAC/g DW), suggesting that phenolic compounds are partially lost during the peeling process, most likely because they are localized in the outer layers of the tuber. As expected, tiger nut oil showed significantly higher antioxidant activity (64.82 ± 2.591 mg TEAC/L; *p* < 0.001) than both tuber samples, indicating that bioactive phenolic compounds are efficiently transferred into the lipid fraction during oil extraction. This enhanced antioxidant capacity may contribute to maintaining of peroxide and acid values, thereby improving the overall oxidative stability of the oil. Owon et al. [68] confirmed the high antioxidant activity of tiger nut tubers, reporting 43.42% and 60.33% DPPH radical scavenging activity at concentrations of 100 μg and 200 μg powder/mL ethanol, respectively. Furthermore, findings from Bosch et al. [69] suggest that the antioxidant properties and TPC of Spanish tiger nuts, particularly organically grown samples, are higher compared to those originating from other regions (e.g., Nigerian conventional samples or samples of unknown origin).

A higher concentration of phenolic compounds is generally associated with greater antioxidant activity [70]. The TPC of tiger nut oil has previously been reported as 16.50 mg GAE/100 g, which is significantly higher than that of sunflower oil (5.0 mg GAE/100 g) [71]. In our study, the TPC of tiger nut oil was 22.81 ± 0.918 mg GAE/L, indicating that its elevated phenolic content contributes directly to its antioxidant potential. Moreover, the notable TPC and antioxidant activity of tiger nut tubers underline their potential as a functional food capable of contributing to the prevention of oxidative stress-related diseases [72]. Owon et al. [68] reported that phenolic extracts from tiger nut tubers contained 197 mg GAE/100 g, with benzoic, salicylic, ellagic acids, and colchicine, identified as predominant phenolic compounds. In our research, the TPC values of Spanish natural tiger nut tubers (2.58 ± 0.428 mg GAE/g DW) were significantly higher than those of peeled tubers (1.71 ± 0.109 mg GAE/g DW) (Table 1), with statistically significant differences observed among all evaluated samples (*p* < 0.01). This finding highlights the peel as the primary reservoir of phenolic compounds. These results are consistent with the findings of Bosch et al. [69], who reported higher TPC values in Spanish tiger nuts (1.80–2.02 mg GAE/g) compared to Nigerian samples (1.52 mg GAE/g).

While the present results show a clear association between TPC and antioxidant activity, further studies are needed to better understand the mechanisms involved. Future research should focus on the quantification of individual bioactive compounds and on evaluating their individual and potential synergistic antioxidant effects in different matrices. This would help to clarify whether the enhanced antioxidant activity of tiger nut oil is mainly driven by the concentration of specific phenolic compounds or by matrix-dependent interactions affecting their bioavailability and effectiveness.

### 3.2. Assessment of Fatty Acids Profiles and Health Benefits Indices

The composition of FAs and health-related lipid indices in tiger nut tubers and oil is presented in Table 2. A total of nine individual FAs were identified in the analyzed samples.

Tiger nut tubers are a good source of monounsaturated, predominantly long-chain FAs (C16–C20), with an FA composition comparable to that of olive oil [73,74]. They exhibit elevated concentrations of oleic acid (56–85%), along with smaller proportions of palmitic (10–20%), linoleic (8.0–12%), and stearic acids (0.30–5.30%) [17], in accordance with the results obtained in the present study. Furthermore, α-linolenic and palmitoleic acids are generally present in negligible concentrations [75], which also aligns with our findings. In the present study, oleic acid was the predominant FA in all evaluated samples. This lipid fraction is known for its protective properties against coronary, autoimmune, and inflammatory disorders, as well as for its antithrombotic effects and its role in regulating blood pressure [76,77]. Oleic acid contributed to the high total MUFA content (Table 2), in agreement with the findings documented by Yu et al. [73] and Zhao et al. [78]. Similarly, the FA composition reported by Hu et al. [79] showed a comparable profile, with slightly higher oleic acid content (75.60%) and lower SFA values. Overall, our results are similar to the values reported by Adel et al. [80]. The percentage of oleic acid has been shown to vary depending on geographical origin. Reported values from Egypt, Ghana, Nigeria, the Eastern Mediterranean region, and Turkey were 69.50%, 65.55%, 76.10%, 72.70%, and 68.92–73.29%, respectively [14,81,82,83,84]. In our study, tiger nut tubers originated from Valencia, and the observed oleic acid content was consistent with samples from Mediterranean areas.

In the study by El-Naggar [9], oleic acid was identified as the major FA in tiger nut oil (65.80%), followed by palmitic (15.40%), linoleic (5.50%), and arachidic (0.61%) acids. In our study, tiger nut oil also exhibited a high proportion of oleic acid (68.29%; *p* < 0.001). Additionally, there were significantly higher levels of palmitic (*p* < 0.05), stearic (*p* < 0.001), linoleic (*p* < 0.001), and arachidic (*p* < 0.001) acids in tiger nut oil compared to tubers. According to the European Food Safety Authority (EFSA) Panel on Nutrition, Novel Foods and Food Allergens [85], tiger nut oil was evaluated and confirmed to meet the safety requirements for classification as a novel food. In 2025, Commission Implementing Regulation (EU) 2025/1528 authorized tiger nut oil for use as an ingredient in various food categories, and it is now included in the European Union list of authorized novel foods pursuant to Regulation (EU) 2015/2283 [86,87].

The SFA content in tiger nut oil is slightly higher than that in olive oil, whereas the concentration of PUFA is lower. Nevertheless, not all molecular species of SFAs influence serum cholesterol equally. C12:0, C14:0, and C16:0 have been shown to increase serum cholesterol concentrations by suppressing low-density lipoprotein receptor (LDLR) activity. In contrast, short-chain SFAs (C4:0, C6:0, C8:0, and C10:0) are rapidly oxidized to acetyl-CoA in the liver and therefore do not adversely affect LDLR function. Stearic acid appears biologically neutral and has no significant effect on circulating LDL-C levels [88]. Tiger nut oil contains a moderate amount of linoleic acid (5–12%), which is lower than that found in sunflower or soybean oil [17,81]. This moderate proportion of linoleic acid, combined with the predominance of oleic acid, contributes to the oxidative stability of tiger nut oil while still providing an essential dietary PUFA involved in membrane structure and lipid metabolism. In the present study, the linoleic acid content in tiger nut oil was slightly higher (10.03%; *p* < 0.001) than in tubers, and this value corresponds to the upper range reported in the literature [17,81]. Notably, not all PUFA classes exert the same effect on CVD prevention. The short-term administration of fish oil, which is abundant in docosahexaenoic acid may influence the functionality of peroxisome proliferator-activated receptor gamma (PPAR-γ) and provide protective effects against atherosclerotic lesions [89]. Clinical investigations further demonstrate that eicosapentaenoic acid supplementation leads to reduced plasma triacylglycerol concentrations and exerts anti-inflammatory and anti-thrombotic effects [90]. Additionally, a detailed narrative review summarizing the available data suggested that the consumption of linoleic acid is associated with a decreased CVD risk; however, more research is needed to explain the underlying mechanisms [91].

At present, there is little information on key health-related lipid indices, including the P/S ratio, AI, TI, and the h/H ratio, in tiger nut samples. The P/S ratio is based on the premise that PUFAs generally lower LDL-C and total serum cholesterol, whereas SFAs tend to increase them; therefore, a higher P/S ratio is associated with more favorable cardiovascular outcomes [92]. According to current dietary guidelines, a P/S ratio above 0.40 is recommended for healthy foods and diets [93,94,95]. The tiger nut samples in this study exhibited P/S ratios between 0.502 and 0.510, with peeled tiger nut tubers showing the highest value (Table 2), indicating a potentially protective effect against cardiovascular risk factors. The AI elucidates the relationship between the cumulative amounts of SFAs and unsaturated FAs. Ulbricht and Southgate [53] introduced AI as an improved indicator of atherogenic potential of FA in comparison with the P/S ratio, which was perceived as overly simplistic. Values of AI < 1.0 are considered nutritionally favorable, and the closer the value is to zero, the lower the risk of atherosclerosis. All of our samples exhibited low AI values (0.159–0.163 for natural or peeled tiger nut tubers; 0.173 for oil), indicating a highly beneficial lipid profile for cardiovascular health (Table 2). For comparison, cold-pressed sunflower and rapeseed oils typically show even lower AI values, around 0.05 [96,97]. The AI reported for olive oil is 0.16, which is slightly lower than the value observed in tiger nut oil. Nut oils display AI values ranging from 0.07 in pecan oil to approximately 0.15 in pistachio oil. In contrast, Amazonian and tropical oils (e.g., 0.20 for para nut oil, 0.30 for tucuma oil) tend to contain higher proportions of SFA, which is associated with increased AI values [96]. The TI, introduced alongside AI, characterizes the thrombogenic potential of FAs, indicating their tendency to promote clot formation in blood vessels. It represents the ratio between pro-thrombogenic (C12:0, C14:0, and C16:0) and anti-thrombogenic FAs (MUFAs and *n*-3 and *n*-6 PUFAs) [53]. In our study, tiger nut tubers (0.412–0.418) exhibited slightly lower TI values than tiger nut oil (0.494; Table 2), supporting their positive role in cardiovascular health. Similar TI values were reported for macadamia nuts (0.44), as well as for avocado oil (0.41) and olive oil (0.39), while higher TI values were observed for pumpkin seed oils (0.47–0.56) [96]. Both AI and TI are widely used to evaluate the nutritional quality of FAs and their potential health effects. Still, to this day, no organization has developed standardized benchmarks for these indices. As research advances, their calculation methods may be refined using expanded lipidomics datasets and computational modeling approaches [92].

Based on the relationship between dietary FAs and the modulation of plasma LDL-C [88], the h/H ratio illustrates the equilibrium between hypocholesterolemic and hypercholesterolemic FAs. In contrast to the P/S ratio, the h/H ratio may more accurately reflect the effect of FA composition on cardiovascular risk. However, similar to the AI and TI, this index might involve more kinds of FAs such as other molecular species of MUFA, with the potential for the assignment of disparate weights to different molecular FA species. A higher h/H ratio is therefore considered more favorable. In our research, the h/H ratio ranged from 6.099 for natural tiger nut tubers to 6.210 for peeled tubers. Tiger nut oil exhibited an h/H ratio of 5.802, which reflects a highly favorable hypocholesterolemic potential. For comparison, the lowest h/H ratios have been reported for coconut oil and ghee butter (0.10 and 0.50, respectively) [98]. The h/H ratio for olive oil was reported to be 6.10 in a study by Hashempour-Baltork et al. [99]. Thus, the h/H ratio of tiger nut oil is substantially higher, placing it among lipid sources with very low atherogenic and thrombogenic potential and demonstrating its nutritional advantage compared with many widely used dietary oils. These findings highlight tiger nut oil as a functionally valuable ingredient with promising applicability across a wide range of food products.

### 3.3. Evaluation of the Mineral Composition and Potential Health Risks Associated with Tiger Nut Consumption

According to several studies, tiger nut tubers are highly nutrient-dense and contain nearly all the elements required for healthy growth and development in children and adults [33]. Samples of dry, raw, and roasted tiger nut tubers have been reported to contain significantly high levels of K, Ca, Na, and Mg [30,72,74], which is in accordance with our results (Table 3). Natural and peeled tiger nut tubers, as well as tiger nut oil, exhibited statistically significant differences in mineral composition for most of the analyzed elements (*p* < 0.001), particularly for K, Ca, Na, and Mg. The presence of these macroelements highlights the nutritional relevance of the studied matrices, as these minerals participate in various metabolic processes of the human body. The overall trend of microelement or trace element levels in tiger nut tuber samples was as follows: Al > Fe > Zn > Cu > Sr > Mn > Li > Ba > Se > As > Cr; < LOD (below the limit of detection): Ag, Cd, Co, Ni, Pb, and Sb.

Iboyi et al. [100] carried out the analysis of mineral elements using atomic absorption spectrophotometry (AAS). According to their results, Ca, Mg, and Se predominated in *Cyperus esculentus*. Ogunlade et al. [30] analyzed raw tubers obtained from Ado-Ekiti (Nigeria) and reported the following mineral profile: Na (101.30 mg/100 g), which is higher than the value observed in our study, as well as K (122.40 mg/100 g), Ca (83.0 mg/100 g), Fe (3.60 mg/100 g), and Mn (0.20 mg/100 g). Our results demonstrated a high concentration of K in natural (4566.96 ± 9.190 mg/kg DW) and peeled (3588.37 ± 9.713 mg/kg DW) tiger nut tubers. The concentration of Na was significantly higher (*p* < 0.001) in natural tiger nut tubers (282.94 ± 0.486 mg/kg DW) compared to peeled tubers (167.80 ± 2.027 mg/kg DW) and oil (84.96 ± 0.356 mg/L). Duman [101] reported higher levels of minerals in yellow and honey tiger nut tubers, with Ca (1159 ± 13.270; 1648 ± 9.850 mg/kg), K (8673 ± 46.110; 7456 ± 28.960 mg/kg), and Fe (85.0 ± 3.280; 104.0 ± 7.560 mg/kg), respectively. On the other hand, the content of Zn (10.30 ± 0.70 and 10.0 ± 1.30 mg/kg) was lower than values detected in our research. Moreover, we achieved a lower Na/K ratio compared to the results of the aforementioned studies, suggesting that tiger nuts may be particularly suitable for inclusion in diets of patients with hypertension or CVD.

Food safety has become a global priority, not only for individual countries but also for international entities, including the European Union and the United Nations [57]. Therefore, health risk assessment indices were applied in the present study. To our knowledge, there are currently only a limited number of studies assessing EDI and potential health risks associated with the consumption of tiger nut tubers or oil. Previously, studies have focused mainly on heavy metals in commercial tiger nut drinks and have generally indicated no appreciable non-carcinogenic risk for consumers [102,103]. Our results obtained from the EDI of individual elements (Table 4) indicate that K appears as the leading macroelement across both groups, with the highest EDI observed in natural tiger nut tubers (0.195074 mg/kg/day). Calcium, which plays a significant role in the mineralization of bones and teeth, also showed higher EDI values in natural tiger nut tubers, corresponding to the increased content of this element (Table 3). The remaining macroelements exhibited lower levels of daily exposure (Table 4). The monitored microelements showed very low EDI values, all of which were lower than the RfD recommended by the US EPA [62]. Iron is a functional component of hemoglobin and several other key compounds involved in respiration, immune function, and cognitive development [104]. In our study, the highest EDI value of Fe was observed in natural tiger nut tubers (0.001320 mg/kg/day). The EDI values for Cu were relatively similar in both groups. Among the trace elements in peeled tiger nut tubers, the highest EDI was recorded for Zn (0.001224 mg/kg/day), which is an essential trace element with a significant role in the immune system, cell division, and DNA synthesis [105]. The higher Zn content in tiger nut tubers may support their potential use as a functional food with a favorable nutritional profile. The concentrations of other elements were substantially below tolerable daily intake limits. Nevertheless, their presence remains of interest from the perspective of biodiversity and the ability of plants to selectively accumulate trace elements.

Target hazard quotient and TTHQ for natural and peeled tiger nut tubers were calculated and presented in Table 5. The overall THQ trend in natural tiger nut tubers was as follows: Cu > Zn > Al > Fe > Li > Sr > Mn > Ba, while in peeled tiger nut tubers it followed the order: Cu > Zn > Fe > Al > Li > Se > Sr > Mn. All THQ values were below the threshold of 1 (Table 5), signifying that consumption of the assessed elements is considered safe and is not expected to pose a significant health risk. Among the monitored elements, Cu (0.008671–0.008753) and Zn (0.004330–0.004082) exhibited the highest individual THQ values. Nevertheless, these values remained well below the risk threshold, suggesting that long-term consumption of either natural or peeled tiger nut tubers in typical amounts does not result in exceeding the levels associated with potential health risk. Aluminum was also detected and included in the THQ evaluation. Its contribution to the overall risk was low, and the THQ values remained significantly below the threshold of concern, indicating no toxicological risk at the observed concentrations. The low cumulative TTHQ values confirm that regular consumption of tiger nut tubers does not pose a health risk associated with simultaneous exposure to multiple elements and supports their safety as a food ingredient.

## 4. Conclusions

The results demonstrate that tiger nut tubers are an important plant source, rich in monounsaturated FAs, particularly oleic acid. Tiger nut tubers also exhibited favorable lipid health indices (PUFA/SFA > 0.50, low AI and TI, high h/H ratio), supporting their potential role in cardiometabolic health. High levels of total phenolic compounds and strong antioxidant activity in tubers and oil further suggest protective effects against oxidative stress-related diseases. Additionally, tiger nut tubers displayed a beneficial mineral profile, particularly high K, Ca, Na, and Mg contents and a low Na/K ratio, reinforcing their suitability for diets targeting hypertension and cardiovascular disease prevention. Moreover, the EDI, THQ, and TTHQ values for elements remained well below established risk thresholds, indicating that regular consumption of tiger nut tubers does not pose toxicological concerns. These findings support the classification of tiger nut oil and its recent authorization as a novel food within the European Union and highlight its applicability as a functionally valuable ingredient with promising potential across a wide range of food products. Future studies should focus on a comprehensive assessment of antioxidant capacity using multiple complementary assays, as well as on detailed phenolic profiling. Additional prospective research could be valuable to evaluate the technological properties and organoleptic attributes of tiger nut-based products using a trained sensory panel, and to assess their antimicrobial activity. These evaluations will help clarify the overall suitability of tiger nut–based products for safe and acceptable use by consumers.

## Figures and Tables

**Table 1:** Results of antioxidant activity and total phenolic content of analyzed samples.

Parameters	Natural Tiger Nut Tubers (mg/kg DW) (x ± SD)	Peeled Tiger Nut Tubers (mg/kg DW) (x ± SD)	Tiger Nut Oil (mg/L DW) (x ± SD)	*p*-Value
Antioxidant activity (TEAC)	2.77 ± 0.201 ^b^	1.49 ± 0.270 ^b^	64.82 ± 2.591 ^a^	*p* < 0.001
Total phenolic content (GAE)	2.58 ± 0.428 ^b^	1.71 ± 0.109 ^c^	22.81 ± 0.918 ^a^	*p* < 0.01

x—arithmetic mean; SD—standard deviation; DW—dry weight; TEAC—Trolox equivalent antioxidant capacity; GAE—gallic acid equivalent (one-way ANOVA followed by Tukey’s multiple comparison test). Different small letters indicate statistically significant differences between samples (one-way ANOVA followed by Tukey’s multiple comparison test; *p* < 0.05).

**Table 2:** Fatty acid composition (%) and health benefits indices of evaluated tiger nut tubers (natural, peeled) and oil, detected by GC-FID.

Notation	Individual of the FAs	Molecular Mass	Natural Tiger Nut Tubers (x ± SD)	Peeled Tiger Nut Tubers (x ± SD)	Tiger Nut Oil (x ± SD)	*p*-Value
C16:0	Palmitic acid	256.43	13.23 ± 0.108 ^b^	13.04 ± 0.124 ^c^	13.55 ± 0.085 ^a^	*p* < 0.05
C16:1	Palmitoleic acid	254.41	0.33 ± 0.001 ^a^	0.31 ± 0.008 ^b^	0.25 ± 0.005 ^c^	*p* < 0.05
C18:0	Stearic acid	284.48	3.90 ± 0.042 ^b^	3.92 ± 0.049 ^b^	6.09 ± 0.170 ^a^	*p* < 0.001
C18:1cis *n*-9	Oleic acid	282.47	71.55 ± 0.161 ^a^	71.79 ± 0.329 ^a^	68.29 ± 0.453 ^b^	*p* < 0.001
C18:2cis *n*-6	Linoleic acid	280.45	8.76 ± 0.077 ^b^	8.78 ± 0.100 ^b^	10.03 ± 0.231 ^a^	*p* < 0.001
C18:3 *n*-3	α-linolenic acid	278.43	0.18 ± 0.002 ^a^	0.19 ± 0.010 ^a^	0.13 ± 0.002 ^b^	*p* < 0.001
C20:0	Arachidic acid	312.53	0.51 ± 0.014 ^b^	0.52 ± 0.008 ^b^	0.60 ± 0.041 ^a^	*p* < 0.001
C20:1 *n*-9	*cis*-11-eicosenoic acid	310.51	0.21 ± 0.001 ^b^	0.22 ± 0.007 ^a^	0.17 ± 0.003 ^c^	*p* < 0.001
C24:0	Lignoceric acid	368.63	0.16 ± 0.005	0.16 ± 0.002	0.17 ± 0.020	*-*
∑SFA			17.80	17.64	20.41	
∑MUFA			72.09	72.33	68.70	
∑PUFA			8.94	8.97	10.15	
**Health Lipid Indices**						
PUFA/SFA			0.502	0.510	0.500	
AI			0.163	0.159	0.173	
TI			0.418	0.412	0.494	
h/H			6.099	6.210	5.802	

x—arithmetic mean; SD—standard deviation; SFA—saturated fatty acid; MUFA—monounsaturated fatty acid; PUFA—polyunsaturated fatty acid; AI—atherogenicity index; TI—thrombogenicity index; h/H—hypo-/Hypercholesterolemic ration (one-way ANOVA followed by Tukey’s multiple comparison test). Different small letters indicate statistically significant differences between samples (one-way ANOVA followed by Tukey’s multiple comparison test; *p* < 0.05).

**Table 3:** Mineral matters analysis of tiger nut samples using ICP-OES.

Elements	Natural Tiger Nut Tubers (mg/kg DW) (x ± SD)	Peeled Tiger Nut Tubers (mg/kg DW) (x ± SD)	Tiger Nut Oil (mg/L) (x ± SD)	*p*-Value
K	4566.96 ± 9.190 ^a^	3588.37 ± 9.713 ^a^	6.44 ± 0.126 ^b^	*p* < 0.001
Ca	756.99 ± 1.573 ^a^	467.71 ± 2.408 ^b^	192.12 ± 0.644 ^c^	*p* < 0.001
Na	282.94 ± 0.486 ^a^	167.80 ± 2.027 ^b^	84.96 ± 0.356 ^c^	*p* < 0.001
Mg	102.59 ± 0.119 ^a^	104.40 ± 1.194 ^a^	4.32 ± 0.014 ^b^	*p* < 0.001
Al	95.18 ± 0.982 ^a^	24.59 ± 1.617 ^c^	0.36 ± 0.174 ^b^	*p* < 0.05
Fe	30.97 ± 0.188 ^a^	17.79 ± 3.044 ^b^	0.13 ± 0.026 ^c^	*p* < 0.001
Zn	30.63 ± 0.366 ^a^	28.67 ± 0.822 ^b^	0.94 ± 0.035 ^c^	*p* < 0.05
Cu	8.12 ± 0.017	8.17 ± 0.306	-	-
Sr	7.16 ± 0.018 ^a^	4.23 ± 0.564 ^b^	1.84 ± 0.005 ^c^	*p* < 0.001
Mn	0.097 ± 0.005 ^b^	0.156 ± 0.004 ^a^	0.048 ± 0.000 ^c^	*p* < 0.001
Li	0.060 ± 0.001 ^a^	0.020 ± 0.011 ^b^	0.001 ± 0.000 ^c^	*p* < 0.05
Ba	0.023 ± 0.002 ^b^	-	0.997 ± 0.002 ^a^	*p* < 0.001
Se	-	0.04 ± 0.000 ^b^	1.21 ± 0.442 ^a^	*p* < 0.001
As	-	-	0.80 ± 0.499	-
Cr	-	-	0.025 ± 0.009	-

x—arithmetic mean; SD—standard deviation (one-way ANOVA followed by Tukey’s multiple comparison test). Different small letters indicate statistically significant differences between samples (one-way ANOVA followed by Tukey’s multiple comparison test; *p* < 0.05).

**Table 4:** Calculated Estimated Daily Intake (EDI) for elements in the analyzed samples (mg/kg/day).

Elements	Natural Tiger Nut Tubers (mg/kg DW)	Peeled Tiger Nut Tubers (mg/kg DW)
K	0.195074	0.153274
Ca	0.032334	0.019977
Na	0.012085	0.007167
Mg	0.004382	0.004459
Al	0.004007	0.001050
Fe	0.001320	0.000759
Zn	0.001300	0.001224
Cu	0.000346	0.000348
Sr	0.000305	0.000180
Mn	0.000004	0.000006
Li	0.000002	0.000000
Se	-	0.000001
Ba	-	-

**Table 5:** Results of target hazard quotient (THQ) and total hazard quotient (TTHQ) in analyzed samples.

Elements	Natural Tiger Nut Tubers	Peeled Tiger Nut Tubers
Target hazard quotient (THQ)		
Cu	0.008671	0.008753
Zn	0.004330	0.004082
Al	0.004079	0.001053
Fe	0.001885	0.001089
Li	0.001280	0.000427
Sr	0.000508	0.000302
Mn	0.000029	0.000047
Ba	0.000004	-
Se	-	0.000341
Ag, Cd, Co, Ni, Pb, Sb	-	-
Total target hazard quotient (TTHQ)	0.020786	0.016094

## Data Availability

The data presented in this study are contained within the article. Further inquiries can be directed to the corresponding author.
